# Differential effects of high fat diet-induced obesity on oocyte mitochondrial functions in inbred and outbred mice

**DOI:** 10.1038/s41598-020-66702-6

**Published:** 2020-06-17

**Authors:** Waleed F. A. Marei, Anouk Smits, Omnia Mohey-Elsaeed, Isabel Pintelon, Daisy Ginneberge, Peter E. J. Bols, Katrien Moerloose, Jo L. M. R. Leroy

**Affiliations:** 1 Gamete Research Centre, University of Antwerp, 2610 Wilrijk, Belgium; 2 Department of Theriogenology, Faculty of Veterinary Medicine, Cairo University, Giza, 12211 Egypt; 3 Department of Cytology and Histology, Faculty of Veterinary Medicine, Cairo University, Giza, 12211 Egypt; 4 Laboratory of Cell Biology & Histology, University of Antwerp, 2610 Wilrijk, Belgium; 5 VIB Center for Inflammation Research, Ghent, Belgium; 6 Department of Biomedical Molecular Biology, Ghent University, Ghent, Belgium

**Keywords:** Reproductive biology, Infertility

## Abstract

Maternal obesity can cause reduced oocyte quality and subfertility. Mitochondrial dysfunction plays a central role here, and most often inbred mouse models are used to study these pathways. We hypothesized that the mouse genetic background can influence the impact of high fat diet (HFD)-induced obesity on oocyte quality. We compared the inbred C57BL/6 (B6) and the outbred Swiss strains after feeding a HFD for 13w. HFD-mice had increased body weight gain, hypercholesterolemia, and increased oocyte lipid droplet (LD) accumulation in both strains. LD distribution was strain-dependent. In Swiss mouse oocytes, HFD significantly increased mitochondrial inner membrane potential (MMP), reactive oxygen species concentrations, mitochondrial ultrastructural abnormalities (by 46.4%), and endoplasmic reticulum (ER) swelling, and decreased mtDNA copy numbers compared with Swiss controls (P < 0.05). Surprisingly, B6-control oocytes exhibited signs of cellular stress compared to the Swiss controls (P < 0.05); upregulated gene expression of ER- and oxidative stress markers, high mitochondrial ultrastructural abnormalities (48.6%) and ER swelling. Consequently, the HFD impact on B6 oocyte quality was less obvious, with 9% higher mitochondrial abnormalities, and no additive effect on MMP and stress marks compared to B6 control (P > 0.1). Interestingly, mtDNA in B6-HFD oocytes was increased suggesting defective mitophagy. In conclusion, we show evidence that the genetic background or inbreeding can affect mitochondrial functions in oocytes and may influence the impact of HFD on oocyte quality. These results should create awareness when choosing and interpreting data obtained from different mouse models before extrapolating to human applications.

## Introduction

The prevalence of obesity and metabolic syndrome is increasing and is currently affecting millions of women in reproductive age worldwide. This has been inextricably linked with infertility. Obese women have a lower chance of spontaneous pregnancy, higher risks of early pregnancy losses and miscarriage^1,2^. In addition, success rates following assisted reproductive treatments are usually lower as compared to normal body mass index (BMI) cohorts^3,4^.

Over the last decade, several studies have correlated obesity with reduced oocyte quality using animal models, and elucidated some of the underlying causative mechanisms^5–9^. High fat diet (HFD) is commonly used to induce obesity in mouse models. This results in hyperlipidemia, systemic lipotoxicity and oxidative stress, which is reflected in the ovarian follicle microenvironment^10^. Mimicking such lipotoxic conditions *in vitro* was shown to inhibit the growth of murine secondary ovarian follicles (during 13 days exposure) and substantially reduced oocyte developmental capacity and the quality of the resulting blastocysts^6^. Importantly, mitochondrial dysfunction clearly plays an important role in the pathogenesis of reduced oocyte quality. HFD increased mitochondrial ultrastructural abnormalities in oocytes, altered mitochondrial inner membrane potential (MMP) and ATP production, and altered mitochondrial biogenesis and mtDNA copy numbers, compared to control diet in mice^5,11–13^. The direction and extent of most of these changes are inconsistent among different studies as discussed later in this manuscript.

Most HFD-induced obese mouse models use the C57BL/6 strain. This is an inbred strain which progressively gain weight^8,11,14^ and develop hyperlipidemia and inflammatory responses when supplemented with HFD. The genetic homogeneity in inbred strains minimizes variability in experimental settings. However, the C57BL/6 strain is characterized by low fertility, small litter size and cannibalism of pups, which makes it unreliable for studies focusing on fertility outcomes. Furthermore, inbreeding increases the risk of genetic drift and persistent undiscovered mutations, which may confound responses to experimental factors^15^. After all, extrapolation of the data and conclusions acquired from inbred models to human physiology is almost impossible. In contrast, outbred strains such as Swiss mice are more fertile and display better nurturing behavior. They are also metabolically sensitive to HFD, and develop hypercholesterolemia, obesity, and insulin resistance^16,17^. However, due to the wide genetic variability, more variation is seen in responses to changes in environmental conditions.

The effect of the genetic background on reproductive parameters has been examined only in a few studies comparing inbred and outbred mice and focusing on sensitivity to hormonal stimulation and oocyte developmental competence^18–21^. However, the potential interplay of genetic background and the response to HFD-induced obesity at the oocyte level has not been described yet.

Interestingly, it has recently been shown that the mitochondrial genetic background modulates bioenergetics and susceptibility to metabolic diseases. By cross insertion of mtDNA from C57BL/6 mice to C3H/HeN mice, Fetterman, *et al*.^22^ showed that mitochondria containing the C57BL/6 mtDNA generate more reactive oxygen species (ROS) and have a higher MMP relative to those having the C3H/HeN mtDNA. Oxidative stress and mitochondrial dysfunction are known to cause serious detrimental consequences on oocyte developmental competence, but it is not known yet whether this detrimental effect may vary depending on the genetic background of the mouse strain studied.

In addition, mitochondria also act as biological sensors of cellular stress and regulate unfolded protein responses (UPR) and signaling cascades to promote cell survival or initiate apoptosis, similar to those regulated by the endoplasmic reticulum (ER)^23^. Recent data suggest that mitochondrial dysfunction may affect UPR signaling in oocytes and embryos following short term *in vitro* exposure to lipotoxic conditions^24^. However, there is no data available about the activation of these mechanisms in oocytes in obese individuals following long-term exposure to oxidative stress or lipotoxicity. These responses may also be strain dependent.

Therefore, we hypothesize that HFD-induced obesity has a differential effect on oocyte quality and mitochondrial functions in the inbred C57BL/6 strain as compared to the outbred Swiss mice. We also hypothesize that in addition to the alteration in the mitochondrial ultrastructure and MMP, HFD may also alter UPR signaling in oocytes. To test these hypotheses, we exposed C57BL/6 (hereafter referred to as “B6”) and “Swiss” mice to a long-term high fat diet (13w) to induce obesity. Weight gain and blood composition were evaluated and related to oocyte lipid content, mitochondrial ultrastructure and function, ROS production, mtDNA copy numbers and UPR-related gene expression.

## Results

### High fat diet and body weight gain

**Figure 1:**
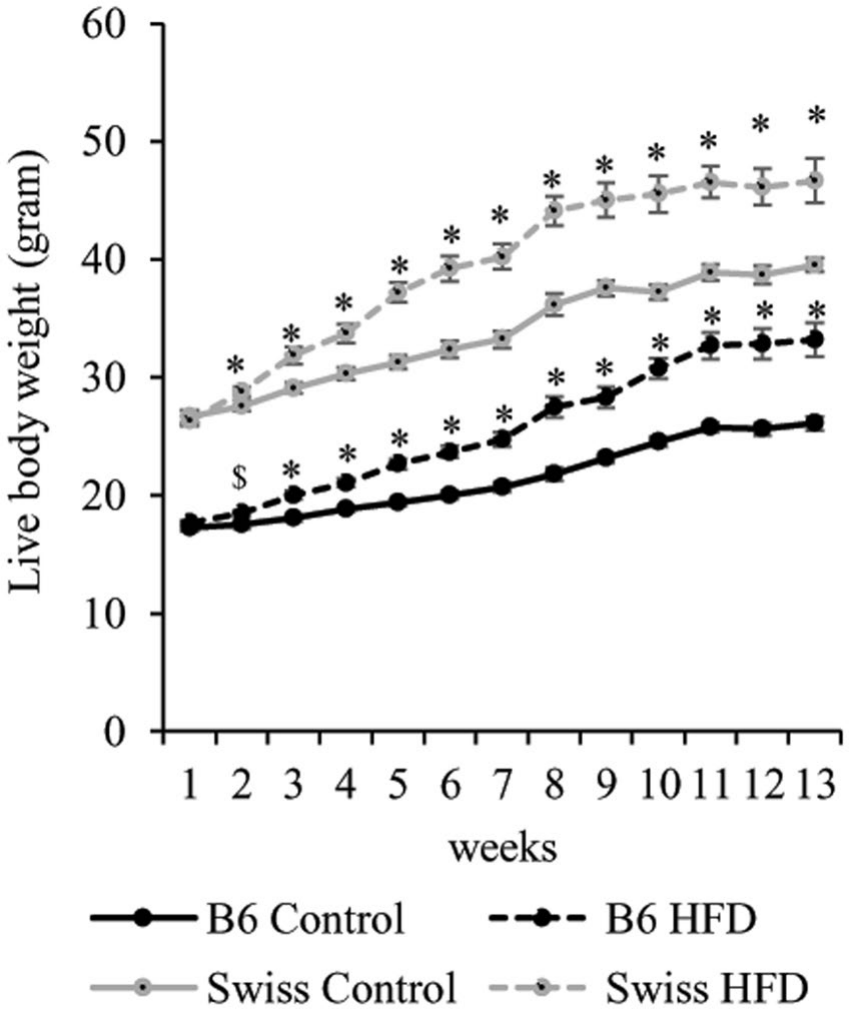
The effect of HFD feeding on body weight gain in B6 and Swiss mice. Data are shown as means ± SEM from 14–16 mice per group. Significant difference (*P* < 0.05) between HFD and Control groups within each strain are indicated by an asterisk (*). Tendencies (0.05 < *P* < 0.1) are indicated by a dollar sign ($).

Swiss and B6 mice fed HFD gained more weight during the 13w-feeding period compared with their corresponding controls of the same strain. Multivariate ANOVA showed that body weight was significantly affected by *Strain* (F = 3277.3, *P* < 0.001), and *Time* (F = 154.1, *P* < 0.01), and their interaction (F = 5.39, *P* < 0.001). The effect of *Diet* (F = 466.8, *P* < 0.001) and the interaction Strain*Diet (F = 9.3, *P* = 0.002) were also significant. Within strain, the increase in weight due to HFD was statistically significant starting from week 2 onwards in the Swiss mice, and from week 3 in the B6 mice (*P* < 0.05) compared to controls. At 13 w, the weight of the Swiss and B6 HFD group was on average 18.1% and 27.2% higher than their corresponding controls (*P* < 0.05), respectively (Fig. 1).

### Effect on blood lipid profile

**Figure 2:**
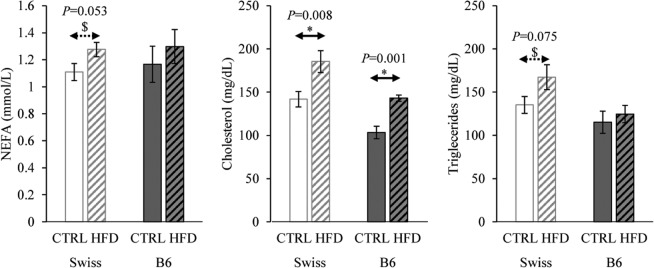
The effect of HFD feeding for 13 weeks on serum lipid profile in B6 and Swiss mice. Data are shown as means ± SEM from 14–16 mice per group. Significant differences (*P* < 0.05) between the groups demarcated by arrows are indicated by an asterisk (*). Tendencies (0.05 < *P* < 0.1) are indicated by a dollar sign ($). Exact *P* values are also shown.

HFD feeding for 13 weeks resulted in a significant increase in blood total cholesterol levels in both Swiss and B6 strains (*P* < 0.05). HFD also resulted in a tendency to increase serum triglycerides (TGs) and NEFAs (*P* < 0.1) in Swiss mice but not in B6 (Fig. 2).

### Effect on oocyte recovery following hormonal stimulation

The average number of oocytes (±SD) recovered from the oviducts following hormonal stimulation tended to be higher in the Swiss control mice compared with the B6 control (14 ± 6.2 vs. 11 ± 3.4 per mouse) however this difference was not statistically significant (*P* = 0.065). HFD did not have any impact on oocyte recovery since the number of oocytes collected from HFD-fed Swiss and B6 were similar to their corresponding controls (13 ± 5.4 and 13 ± 5.9 respectively, *P* > 0.1).

### Effect on intracellular lipid droplets (LD) in oocytes

**Figure 3:**
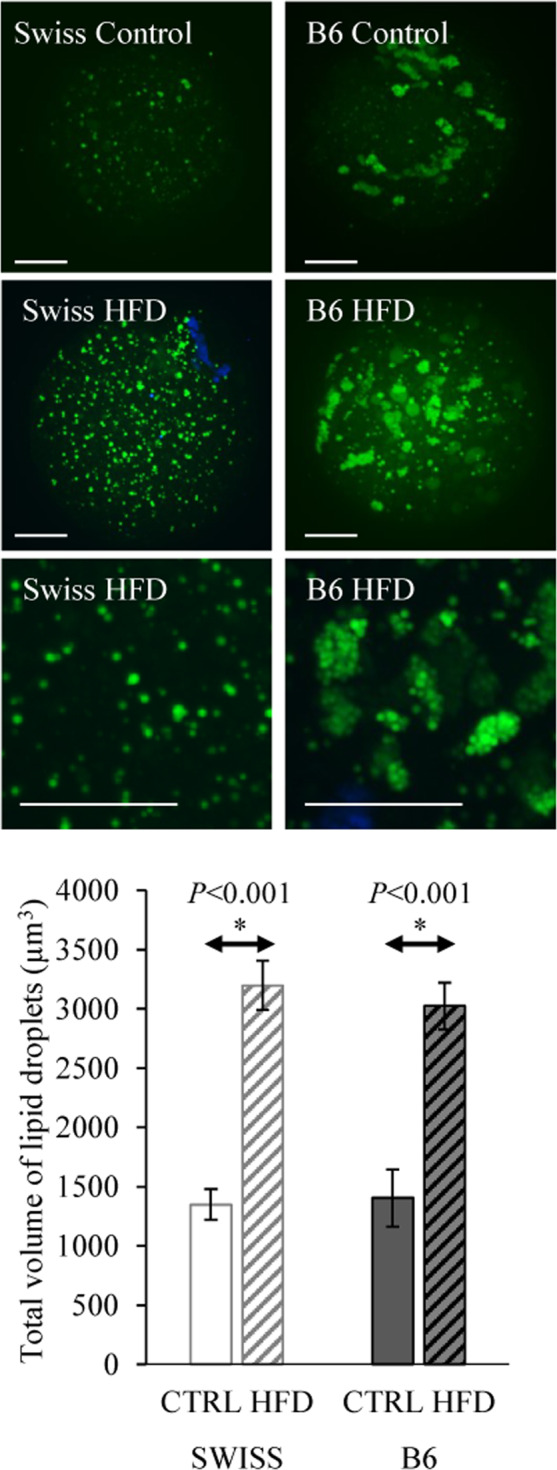
Representative confocal microscope images after BODIPY 493/503 and DAPI staining showing lipid droplets (Green) in oocytes collected from Swiss and B6 mice fed either control or HFD. Each image is a z-stack projection of 40 ×1 µm steps. Scale bar = 17 µm. Data are presented as means ± SEM from one oocyte per animal and 10–12 oocytes per group. Significant differences (*P* < 0.05) between the groups demarcated by arrows are indicated by an asterisk (*).

Lipid droplets in oocytes were examined using BODIPY 493/503 staining and confocal microscopy. Regardless of the type of feeding, the distribution pattern of LDs in oocytes was strain dependent. In Swiss mouse oocytes, LDs were either not clustered or formed very small aggregates and were evenly distributed throughout the ooplasm. Whereas in B6 oocytes, LDs were mostly found in large aggregated clusters (Fig. 3). Quantification of the z-stacks (from a total of 40 oocytes) showed that HFD markedly increased the average total volume of LDs in oocytes compared to controls (*P* < 0.001) in both mouse strains.

### Effect on mitochondrial activity and ROS in oocytes

**Figure 4:**
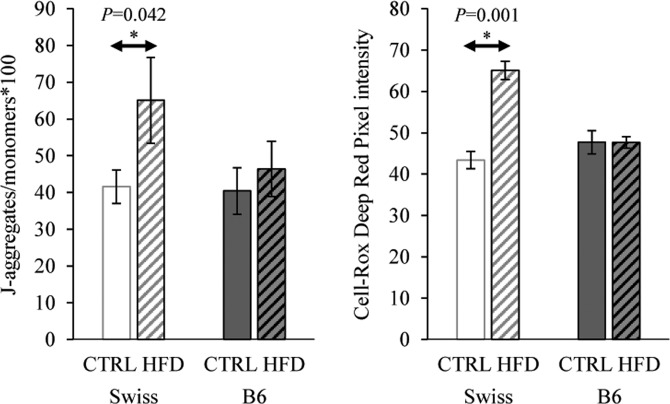
Quantification of mitochondrial activity (JC-1 staining, 590/525 nm intensity × 100) and ROS levels (CellROX Deep Red staining, 660 nm intensity) in B6 (n = 50) and Swiss (n = 67) oocytes from the control and HFD groups. Data are presented as means ± SEM from 1–2 oocytes per animal and 14–16 animals per group. Significant differences (*P* < 0.05) between the groups demarcated by arrows are indicated by an asterisk (*).

Swiss HFD oocytes exhibited higher MMP compared to Swiss controls (*P* < 0.05). This was estimated as the ratio of the J-aggregates (590 nm) to the monomers (525 nm) after JC-1 staining and confocal microscopy (Fig. 4). An increased 660 nm intensity of CellROX Deep Red staining was also evident in the HFD Swiss oocytes indicating higher intracellular ROS concentrations compared with Swiss controls (*P* < 0.05). In contrast, the overall MMP and ROS levels were similar in B6-HFD and B6-control oocytes (*P* > 0.1).

### Effect on mitochondrial ultrastructure in cumulus cells and oocytes

Cumulus oocyte complexes (COCs) were examined with transmission electron microscopy (TEM). In the oocytes, mitochondria were considered to be normal if they were spherical and electron dense, with or without regular vacuoles^25,26^. Mitochondria with abnormal morphology were either vacuolated with loose membranous structures, elongated, dumbbell and rose petal in shape, contained highly electron dense foci or were degenerated (Supplementary Fig. 1).

**Figure 5:**
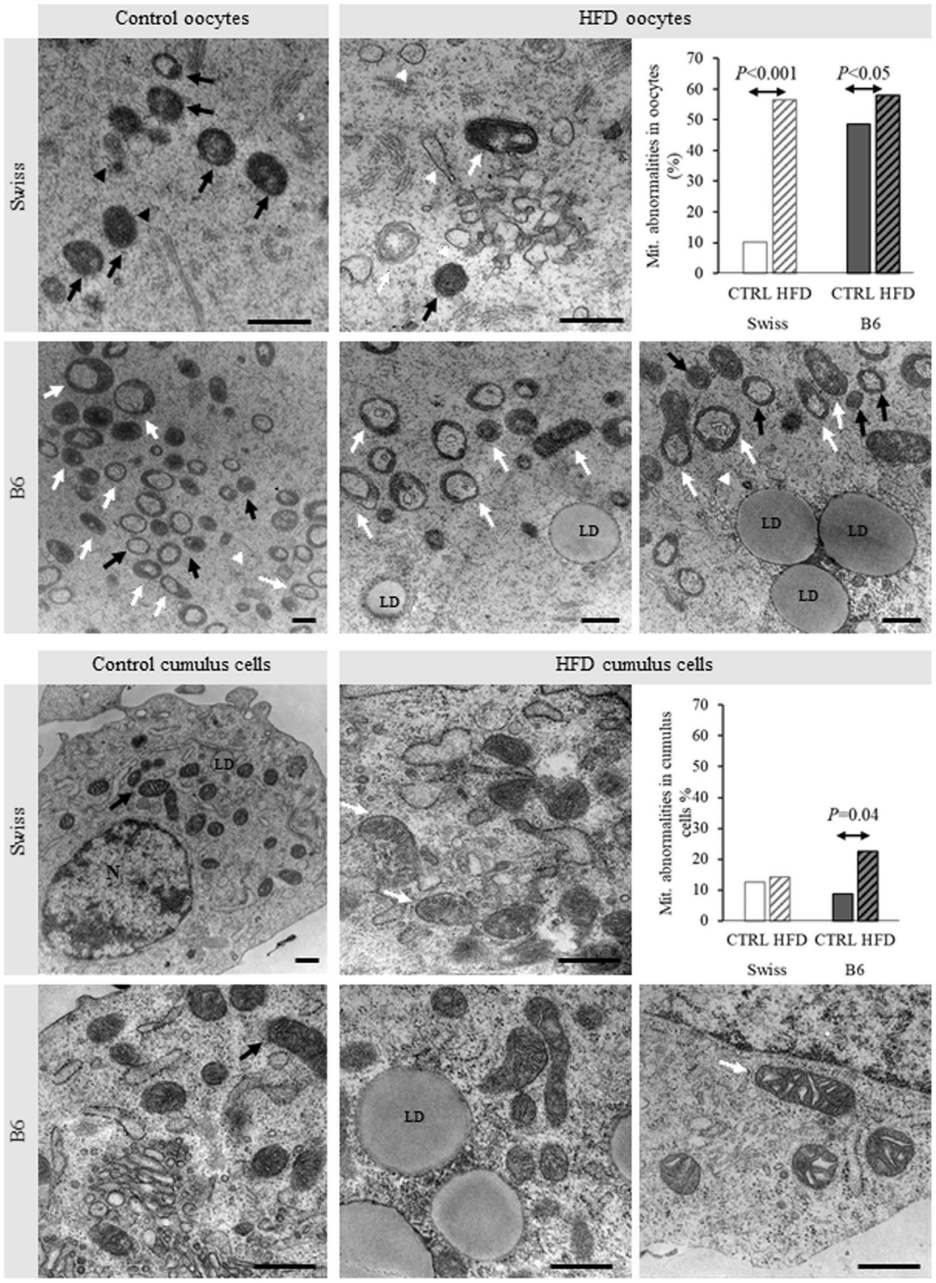
Representative TEM micrographs showing the ultrastructure of mitochondria (arrows) and ER (arrows heads) in oocytes and cumulus cells collected form Swiss and B6 mice fed a control or high fat diet (HFD) for 13 w. Different forms of normal (black arrows) and abnormal (white arrows) mitochondrial structures can be observed as classified in Supplementary Fig. 1. A quantification of the percentage of mitochondrial abnormalities is also included. Data are presented as proportion percentages from 229, 188, 344, and 746 mitochondria counted in Swiss Control, Swiss HFD, B6 Control and B6 HFD respectively, from 3 COCs (from 3 animals) per group. The *P* values between the groups indicated by the arrows are shown.

In Swiss control oocytes, 89.95% of the mitochondria were normal, 18.3% of which contained regular vacuolated areas (Supplementary Table 1). HFD increased the percentage of mitochondrial abnormalities to 56.4%. In contrast, B6-control oocytes exhibited significantly higher proportion of abnormal mitochondria (48.6%) when compared to Swiss controls (*P* < 0.05). The proportion of mitochondrial abnormalities in B6-HFD (57.9%) was about 9% higher than in B6 control (*P* < 0.05) (Fig. 5). A detailed quantification of these mitochondrial abnormalities is shown in “Supplementary Fig. 1” and “Supplementary Table 1”.

In cumulus cells, normal elongated mitochondria with many well-developed cristae could be observed in Swiss and B6 controls. In the B6-HFD group, a significantly higher proportion of mitochondria had distorted shapes and small vacuoles compared to B6-control (*P* < 0.05). In both B6 and Swiss HFD groups, it was noticed that many mitochondria had a relatively lower electron density, which was difficult to quantify.

**Figure 6:**
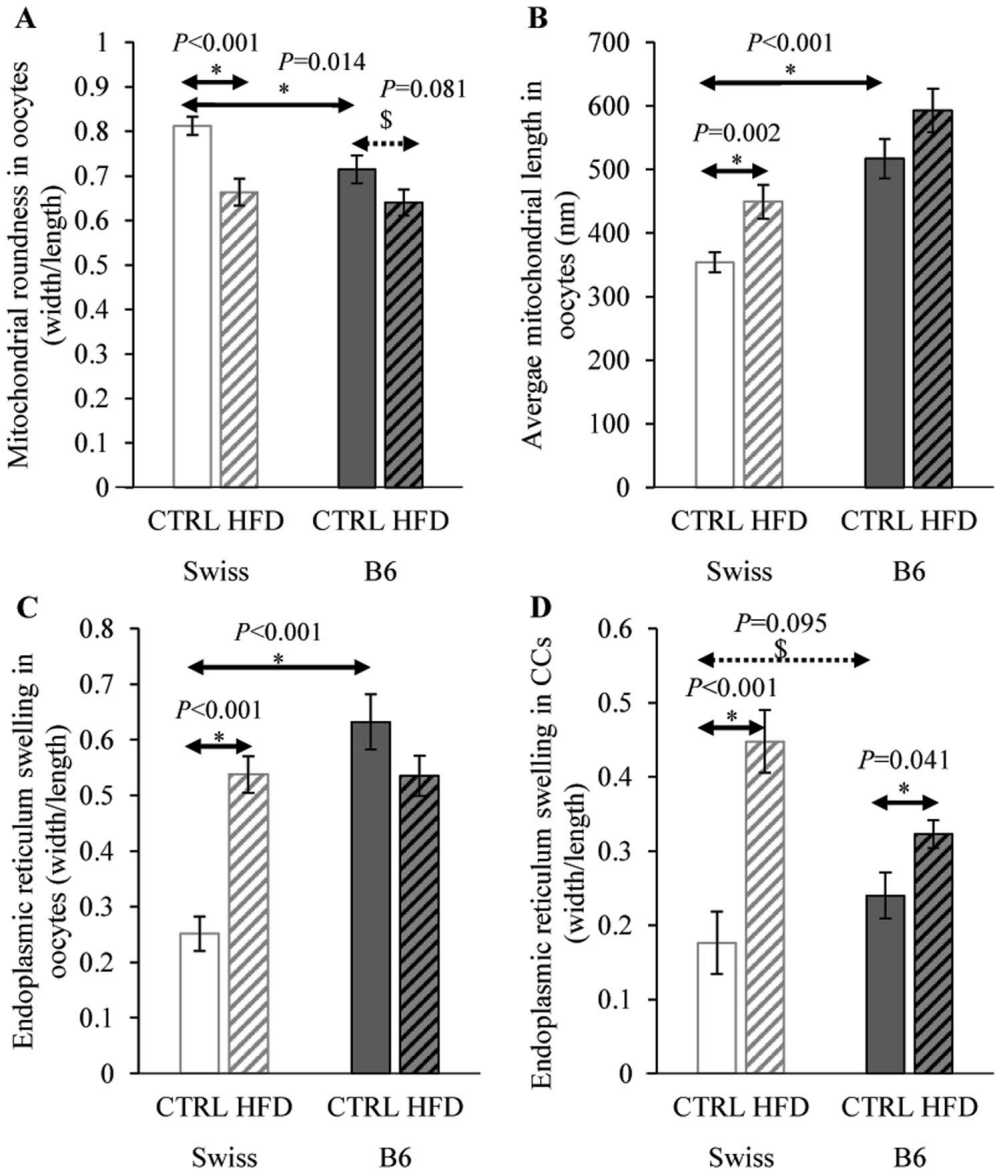
Measurements of the dimensions of mitochondria in oocytes, and ER in oocytes and cumulus cells to estimate the effect of HFD feeding on their ultrastructural morphology in Swiss and B6 mice. Mitochondrial roundness and ER swelling indices were calculated and compared. Data are presented as means ± SEM from the measured organelles in 3 COCs (from 3 animals) per group. Significant differences (*P* < 0.05) between the groups demarcated by arrows are indicated by an asterisk (*). Tendencies (0.05 < *P* < 0.1) are indicated by a dollar sign ($). Exact *P* values are also shown.

To acquire more insight about the impact of HFD, the dimensions of the mitochondria and ER in the TEM images were measured using Image-J software. The mitochondria were found to be almost spherical in the Swiss control oocytes, and less round in the B6 control oocytes (*P* < 0.05) (Fig. 6A). Mitochondrial roundness was further reduced by HFD in both strains (*P* < 0.05). The average length of the mitochondria was shortest in Swiss control oocytes and was significantly longer in Swiss-HFD oocytes and in both B6 groups (*P* < 0.05) (Fig. 6B).

ER swelling, calculated as a ratio between ER width and length was lowest in Swiss control oocytes and cumulus cells and was significantly increased in Swiss HFD (*P* < 0.05). ER swelling was significantly higher in B6 control and HFD oocytes compared to Swiss controls (*P* < 0.05) (Fig. 6C,D).

### mRNA expression of markers of cellular stress in cumulus cells and oocytes

**Figure 7:**
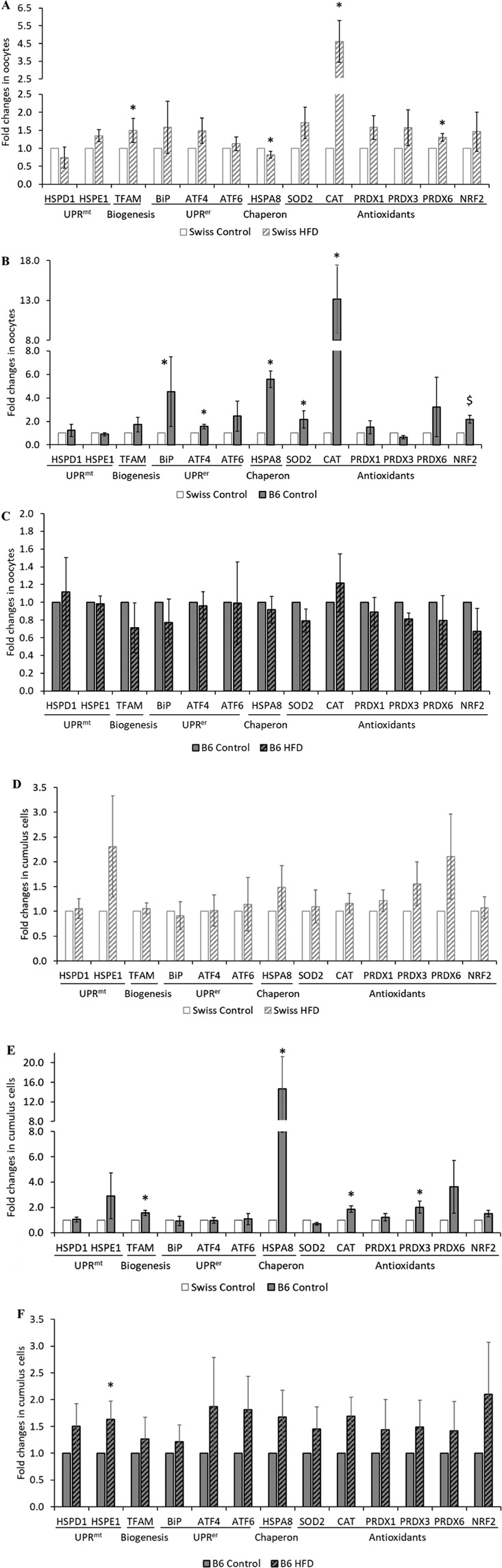
Strain and HFD effects on transcription markers of cellular stress in oocytes (**A**–**C**) and cumulus cells (**D**–**F**). Marker genes related to mitochondrial and ER UPRs and redox regulatory mechanisms were analyzed in B6 and Swiss mice fed either control or HFD. Data were compared within strain or between the controls of the two strains. Columns display means ± SEM of fold changes relative to housekeeping genes from 4 replicates (pools from 3–4 animals per group each). Significant differences are indicated with an astrisk (*P* < 0.05). Tendencies are indicated by a dollar sign “$” (*P* < 0.1 and >0.05).

In Swiss oocytes, HFD increased *CAT* and *PRDX6* mRNA expression. The expression of other tested genes related to redox regulatory mechanisms (*SOD2*, *PRDX1*, *PRDX3*, *NRF2*) were not significantly different from controls. Importantly, the expression levels of markers of mitochondrial UPRs (*HSPE1* and *HSPD1*) and ER UPRs (*BiP*, *ATF4*, and *ATF6*) in the Swiss HFD oocytes were also similar to the Swiss controls (*P* > 0.1). Nevertheless, *TFAM* expression was higher in the HFD group (*P* < 0.05). *HSPA8* was significantly lower with *P* < 0.05 but the relative difference in fold change was very subtle compared to the controls (Fig. 7A).

Surprisingly, several oxidative stress related transcripts were upregulated in control B6 oocytes when compared to the Swiss control group (Fig. 7B). This includes an increased expression of *CAT*, *SOD2*, and *HSPA8* (*P* < 0.05), and a tendency to higher *NRF2* transcript levels (*P* < 0.1). ER stress-related transcripts were also upregulated (*ATF4* and *BiP*), whereas mitochondrial UPR-related genes were not differently expressed. HFD did not induce any further difference in the expression level of any of the tested genes in the B6 oocytes (*P* > 0.01) (Fig. 7C).

In cumulus cells, the variability across replicates was high in the Swiss HFD group, and despite numerical increase in some stress related genes, none of them were statistically significant compared to the controls (*P* > 0.1) (Fig. 7D). However, as observed in the oocytes, cumulus cells from B6 controls showed a significantly higher expression of *CAT*, *HSPA8* and *PRDX3*, as well as *TFAM*, compared with the Swiss control (*P* < 0.05) (Fig. 7E). Markers of mitochondrial and ER UPRs were not altered in B6 controls, but the mitochondrial *HSPE1* was upregulated in response to HFD in the B6 cumulus cells compared to B6 controls (*P* < 0.05) (Fig. 7F).

### Effect on mtDNA copy numbers in oocytes

**Figure 8:**
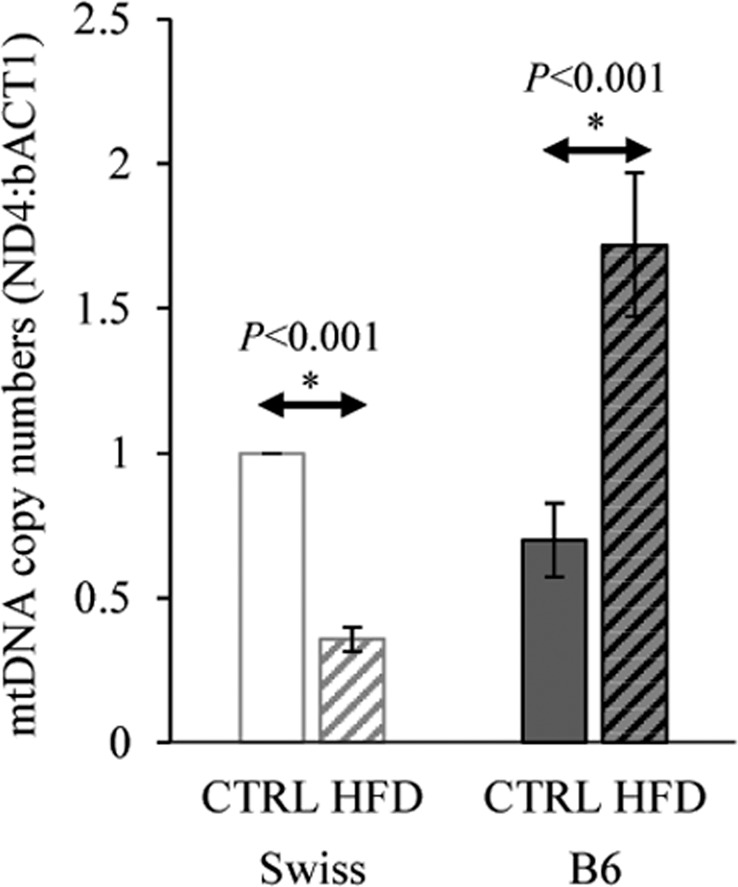
Alteration in mtDNA copy numbers in oocytes collected from Swiss and B6 mice fed a control or high fat diet (HFD) for 13 w. Data are shown as mean ± SEM from 3 replicates (pools from 3–4 animals per group each). Significant differences (*P* < 0.05) between the groups demarcated by arrows are indicated by an asterisk (*).

mtDNA copy numbers in the control oocytes of B6 and Swiss were similar (P > 0.1). HFD reduced mtDNA copy numbers by 2.81X in Swiss oocytes but resulted in a 2.46X increase in mtDNA copy numbers in B6 oocytes (*P* < 0.05 compared to the corresponding control) (Fig. 8).

## Discussion

This study aimed to investigate mitochondrial dysfunction in oocytes at the structural, functional and molecular level in response to HFD-induced obesity. We studied if inbreeding might influence these alterations. Interestingly, we observed major differences in mitochondrial structure in the inbred B6 oocytes as compared to the outbred Swiss oocytes. This was associated with contrasting responses to HFD-induced metabolic stress. We found that feeding HFD for 13 w resulted in various alterations in mitochondrial abnormalities, MMP, mtDNA copy number and cellular stress signaling in oocytes. These effects were highly dependent on the strain.

As expected, HFD increased weight gain and serum total cholesterol concentrations in both strains, which validates the induction of the required metabolic alteration. Swiss mice also exhibited a tendency to increase blood NEFAs and TGs (not statistically significant at *P* < 0.05) while this was not evident in B6 mice. Similar increase in serum cholesterol was described in other studies in C57BL/6 after feeding a HFD, with^27^ or without a concomitant increase in TGs and NEFAs^28^. The impact of this metabolic stress on oocyte quality focusing on mitochondrial functions was the main focus of this study.

There is a strong evidence that hyperlipidemia is linked with reduced oocyte quality, which is mainly due to a direct impact of altered lipid composition of the follicular fluid^29^. Here, we found that HFD increased intracellular lipid accumulation in the Swiss and B6 oocytes. Similar results were reported in CBA and C57Bl/6 J mice^10,12^. This can be due to active lipid accumulation, since murine oocytes can directly incorporate free fatty acids (FFAs) from their microenvironment as demonstrated *in vitro* following direct exposure to FFA-rich media^30^.

Interestingly, we noticed that the LDs distribution was markedly different in B6 and Swiss strains. Lipid droplets were clustered in large aggregates in B6 but were finely scattered in the Swiss ooplasm. Reynolds, *et al*.^10^ showed that after 6 weeks of HFD in C57BL/6 the LDs in the oocytes increased in number and became aggregated. This aggregation was persistent even after 8w of subsequent diet normalization. We suggest that LD clustering could be an important phenomenon that may have extended detrimental effects on oocyte quality, however, this notion has not been tested. Here, the aggregated LDs in B6 mice were also noticed in the control oocytes, at a lower volume, showing that this could be a strain effect and not only induced by diet. LD clustering may be caused by deficiency in  proteins required for LD stabilization^31^. In contrast, smaller LD aggregates (as in the Swiss strain) might provide more surface area for LD‐associated proteins, such as lipases^31^, and may facilitate LD interaction with other organelles, mainly the mitochondria and ER. The impact of LD clustering on oocyte quality requires further investigation.

Accumulation of LD in non-adipose tissue is a cellular mechanism that prevents lipotoxicity by storing intracellular lipids in a neutral status^32^. However, under lipotoxic conditions, upregulated lipolysis and lipophagy may result in a nutritive overload, which affects mitochondrial functions, disrupt cellular metabolism, and may induce oxidative stress and apoptosis^33^. In the present study, we found that MMP (JC-1 aggregates) was significantly increased by HFD in Swiss oocytes, but not in the B6. Higher MMP indicates higher mitochondrial activity, possibly due to increased FA β-oxidation. This could be facilitated by the small LD size in Swiss oocytes, while LD clustering in B6 oocytes may render them less consumable.

Changes in mitochondrial activity in oocytes in response to HFD are inconsistent among different studies. Igosheva, *et al*.^11^ reported that HFD significantly increased MMP in oocytes and zygotes collected from obese C57BL/6 mice. In contrast, Wu, *et al*.^13^ showed that HFD rather reduced mitochondrial activity in CBA mouse oocytes. In addition, the interpretation of relative changes in MMP can also be perplexing. Lower MMP in oocytes, e.g. due to maternal aging, was associated with lower ATP production and reduced developmental competence, whereas higher MMP has been linked with higher embryo development^34^. In contrast, high MMP can also be detrimental, and was linked with increased embryonic arrest at the 2-cell stage in mice and with high fragmentation rate in 8-cell embryos in humans^35^. Interestingly, the increase in MMP at the final stage of IVM in bovine oocytes due to lipotoxicity and oxidative stress has been shown to be temporary, and was followed by a subsequent reduction in MMP (uncoupling) during early cleavage stages^24,36^. Uncoupling occurs particularly in mitochondria that suffer most from high ROS accumulation, which may result in a heterogeneous population of hyperactive and inactive mitochondria within the same oocyte. Therefore overall changes in the MMP in oocytes should always be carefully interpreted in the context of other related parameters and considering the associated ROS levels.

HFD-Swiss oocytes exhibited higher MMP and also higher intracellular ROS as shown using CellROX Deep Red staining. In contrast, HFD did not influence MMP nor ROS in B6 oocytes compared to B6 control. This may either imply that HFD did not elicit an oxidative stress in B6 oocytes, or that the B6 control oocytes already exhibited high levels of oxidative stress for other reasons. The notion that B6 control oocytes also suffer from oxidative stress was further supported by the gene expression results and other outcome parameters as discussed below.

B6 control oocytes had higher *CAT*, *SOD2*, and *NRF2* mRNA expression compared to Swiss control oocytes. The expression of these genes was equally high in B6-HFD oocytes compared to B6 controls. Similar strain dependent differences in some cellular stress related genes were also observed in the B6-control cumulus cells compared to Swiss controls. On the other hand, Swiss HFD oocytes clearly exhibited a significantly higher expression of genes related to Redox regulation compared to Swiss controls.

Looking further into the ultrastructural morphology of the oocytes, we found that the majority (about 90%) of the mitochondria in the Swiss control oocytes had normal ultrastructure^25,26^. As expected, HFD significantly increased mitochondrial ultrastructural abnormalities (about 60%) in Swiss oocytes. Surprisingly, B6 control oocytes already exhibited very high rates of mitochondrial abnormalities. This feature has not been previously brought into attention. However, in Boudoures, *et al*.^27^ we noticed that only 60% of the mitochondria were reported to be round in control C57BL/6 oocytes, while 40% were categorized as elliptical or dumbbell in shape. Moreover, 20% of the spherical mitochondria were described as rose petal in shape^27^. This relatively high percentage of abnormalities was suggested to be probably attributed to the sedentary type of housing of these mice^27^. Here, the housing was the same for both strains showing that the mitochondrial abnormalities in B6 could rather be genetically driven or strain-specific. Feeding HFD for 4 weeks^37^ or for 12 w^27^ resulted in an additional 10–20% increase in the proportions of abnormal mitochondria in B6 oocytes compared to controls, an effect that was also observed here in HFD-B6 (9.36% higher than B6 controls). This effect was much bigger in the Swiss strain.

Additionally, our data clearly show that the roundness of the mitochondria was highest in Swiss control oocytes and significantly lower in B6 control oocytes, and was further reduced by HFD in both strains. Mitochondrial elongation can be an indication of the activation of mitochondrial fission and fusion^38^ and could therefore be a part of a compensatory reparative mechanism. An increased expression of PGC1α and DRP1, which play an important function in mitochondrial biogenesis, has been described in B6 oocytes in response to HFD^37^. Mitochondrial elongation and reduction in electron density normally occur after the 8-cell stage and become more evident at blastocyst formation^39^, coinciding with the change in the metabolic activity of the mitochondria towards an increased glucose utilization and oxygen consumption^40^. Therefore, detection of these changes in HFD oocytes here may indicate a premature alteration in cellular metabolism. Similar ultrastructural abnormalities have been linked with abnormal or delayed meiotic progression of the affected oocytes and abnormal spindle morphology^27^ thus signifying a reduced oocyte developmental competence. Finally, increased ROS production inside the mitochondria could induce mitochondrial fission^41^ which may link the increased mitochondrial elongation and the disruption in Redox regulatory mechanisms observed here in the Swiss HFD oocytes.

ER swelling was increased in the HFD Swiss oocytes, and interestingly in both HFD and control B6 oocytes, compared to Swiss controls. Again, this suggests that the B6 oocytes exhibit high levels of cellular stress regardless of the HFD. ER swelling is an indication of increased ER stress, which is closely related to the mitochondrial dysfunction and increased risk of apoptosis^42^. ER stress has a well described role in mediating the effects of altered lipid metabolism and lipotoxicity in somatic cells. This was also documented in bovine COCs exposed to lipotoxic concentrations of FFAs *in vitro*^43,44^.

Another main aim of the present study was to determine if UPRs occur in the oocytes in response to HFD. Mitochondrial and ER stress in somatic cells are known to stimulate transcription of chaperons and other related factors to control protein misfolding and enhance cell survival^23,45^. Therefore, we examined the transcription levels of marker genes for UPR^er^ (*BiP*, *ATF4* and *ATF6*), and UPR^mt^ (*HSPD1* and *HSPE1*). Keeping in mind the limited transcriptional capacity in oocytes specially at the final stage of maturation, we found that the oxidative stress-related mRNA expression of *CAT* and *PRDX6* were significantly increased in the Swiss HFD group. This is in line with the increased oxidative stress levels illustrated by CellROX Deep Red staining, and suggests that the long-term exposure to HFD during oocyte growth could influence gene expression levels while the oocyte is still transcriptionally active. Nevertheless, we could not detect any corresponding change in UPR related genes despite the evident mitochondrial dysfunction and ER swelling. Perhaps, these responses are not fully functional in mature oocytes at the transcriptional level. We have previously shown that exposure of bovine COCs to lipotoxic concentrations during IVM resulted in several UPR-related proteomic changes^24^, which were persistent until the blastocyst stage^36^. Interestingly, we observed that *BiP* and *ATF4*, as well as *SOD2* and *CAT* were significantly higher in B6 control oocytes compared to the Swiss controls, and were equally high in the B6 HFD group. This is in line with the observed ER swelling and further suggests that these UPRs could also be strain dependent.

Finally it was also important to assess the alteration in mtDNA copy numbers in oocytes in response to HFD. This parameter is linked to mitochondrial dysfunction and oxidative stress. Swiss HFD oocytes exhibited a significantly lower mtDNA, which was associated with a (compensatory) increase in TFAM expression compared to Swiss controls. This could be simply due to mitophagy and removal of damaged mitochondria. In contrast, B6-HFD oocytes had a significantly higher mtDNA content with no difference in TFAM expression compared to B6 controls. A previous study showed a similar increase in mtDNA copy numbers (with a higher TFAM expression) in B6 mice exposed to obesogenic diet compared to lean mice^11^.

The number of mtDNA copies in oocytes increases during folliculogenesis and reaches its maximum at the end of oocyte maturation^46^ thus it may be influenced during long-term exposure to altered follicular environment due to HFD. Short exposure to lipotoxic conditions during IVM (24 h) did not influence mtDNA copy numbers in bovine oocytes despite the evident oxidative stress and mitochondrial dysfunction^36^. The mtDNA copy number in an oocyte is a product of a turnover process during which mitochondrial biosynthesis and degradation take place. Therefore, the increased mtDNA copies in HFD-B6, considering the evident mitochondrial ultrastructural damage, may not necessarily only be due to increased mitochondrial biogenesis, but could also be due to defective mitophagy^47^. This hypothesis requires further investigation. Inhibition of mitophagy using a proteasome inhibitor has been shown to increase mtDNA content in oocytes, which shows that degradation is an important determinant under certain conditions^48^. Human embryos with relatively high mtDNA copy numbers (MitoScore) have significantly higher risk of failure of implantation^49^. Therefore, the increased mtDNA in B6 HFD oocytes could be detrimental at later stages of development.

Mechanisms regulating mitophagy and mitochondrial biogenesis in oocytes and early embryo development have not been clearly elucidated. Recent studies illustrated that accumulation of damaged mitochondria in obesity-exposed oocytes and their transmission to next generations is due to defective mitophagy^26,50^. These studies were carried out using C57BL/6 mice. However, our results strongly suggest that mitochondrial damage and mitophagy may vary in different strains of mice, possibly due to inbreeding. This is crucial for the correct extrapolation of information described in transgenerational studies based on the C57BL/6 strain.

In conclusion, different important aspects of mitochondrial dysfunction in response to HFD-induced obesity appear to be influenced by the genetic background of the mouse model used, including alteration in MMP, mitochondrial ultrastructural abnormalities and alteration in mtDNA copy numbers (and possibly mechanisms regulating mitochondrial biogenesis and mitophagy). This may be caused by inbreeding. Mitochondrial chaperons in oocytes were not affected by long-term HFD at the transcription level in both B6 and Swiss mice. However, B6 oocytes exhibited some upregulated ER UPR markers also suggesting a strain effect. These results should further build on awareness when designing mouse models to study human fertility and transgenerational effects. Depending on the strain used, conclusions can be significantly different. We showed that the outbred Swiss mouse seems to be more sensitive to HFD-induced reduction in oocyte quality when compared to the inbred B6 mouse. Whereas the B6 oocytes appear to be of low quality regardless of the type of diet, which may explain the reduced fertility observed in this strain.

## Materials and Methods

### Animals, diet and experimental design

All procedures in this study were approved by the ethical committee of the University of Antwerp and performed accordingly (ECD nr. 2014/57), and all methods were performed in accordance with the relevant guidelines and regulations. Seven-week-old female outbred Rj:Orl Swiss (n = 32, hereafter referred to as “Swiss”) and inbred C57BL/6 N (n = 32, hereafter referred to as “B6”) mice (Janvier labs) were used. Mice of each strain were randomly divided into two main groups with ad libitum access to either a control (D12450J, Research Diets) or a high fat diet (HFD; D12492, Research Diets) for a period of thirteen weeks. The HFD was composed of 60 kcal% fat from lard, 20 kcal% carbohydrate and 20 kcal% protein. The matched, purified control diet contained 10 kcal% fat from lard, 70 kcal% carbohydrate and 20 kcal% protein. The energy density of these diets were 5.21 kcal/g and 3.82 kcal/g, respectively. Access to water was provided ad libitum. Mice were put on the diet in subgroups of 3–4 animals per treatment (4 replicates) with an interval of few days between replicates to facilitate handling and sample collection. Mice were weighed weekly. Two B6-HFD and one B6-Control mice developed disease symptoms (eye inflammation and skin lesions) and were excluded from the experiment because their food intake was affected.

At 13 weeks, mice received intraperitoneal injections of 10 IU equine chorionic gonadotropin (eCG, Synchrostim**;** Ceva Santé Animale) followed, 48 h later, by 10 IU human chorionic gonadotropin (hCG, Pregnyl; Organon) to induce and synchronize ovulations. Mice were sacrificed 13–14 h after hCG injection by decapitation to allow collection of the maximal volume of blood. After euthanasia, oocytes were collected and further examined as described below.

### Oocyte and cumulus cell collection and preparation for subsequent analyses

*In vivo* matured oocytes were obtained from the oviducts immediately after euthanasia. Each oviduct was dissected together with the ovary and a part of the uterine horn and transferred to a collection tube containing L15 medium (Thermofisher Scientific) supplemented with 50 IU/mL penicillin G sodium salt, and 10% Fetal Bovine Serum.

The cumulus oocyte complexes (COCs) collected from both oviducts of the same animal were pooled. According to the total number of COCs available, COCs from each mouse were distributed for downstream analysis as follows: one whole COC per mouse was fixed in glutaraldehyde solution for transmission electron microscopy (TEM). The remaining COCs were completely denuded by repeated pipetting through 100 µm Stripper tips fitted on EZ-grip (Origio) in a droplet of L15 medium supplemented with 0.3 mg/mL hyaluronidase. Denuded oocytes were transferred to a fresh drop. Two denuded oocytes (per mouse) were immediately transferred to JC-1 and CellROX Deep Red staining to determine mitochondrial activity and intracellular ROS content. One to two oocytes per mouse were fixed in paraformaldehyde 4% for determination of lipid droplet content. The remaining oocytes from each subgroup (n > 15 oocytes from 3–4 animals) were pooled and washed in PBS containing 1 mg/mL PVP and snap frozen in a 1.5 mL tube in a minimum volume for simultaneous RNA and DNA extraction for qPCR and determination of mtDNA content. Meanwhile, the droplets containing the detached cumulus cells from all animals in each subgroup were also immediately pooled, centrifuged, washed in PBS-PVP and snap frozen. All frozen samples were stored at −80 °C until further analyses. Some oocytes were lost during mounting. DNA extraction of 1 replicate failed due to technical reasons. The final numbers of animals used to collect data for each outcome parameter are shown in figure legends.

### Serum collection and analyses

Blood was centrifuged 30 min after collection at 2000 rpm for 10 min, and serum was stored at −80 °C. Serum analyses were performed in a commercial laboratory (Algemeen Medisch Labo, Antwerp, Belgium). Non-esterified fatty acid (NEFA) concentrations were determined using a colorimetric assay (Randox Laboratories Ltd, Crumlin, Co. Antrim, United Kingdom) on an IDS iSYS multi-discipline automated instrument (Immunodiagnostic Systems Hld, Tyne & Wear, UK). Triglycerides (TG) and Cholesterol were measured on an Abbott Architect c16000 (Abbott, Illinois, U.S.A).

### Assessment of oocyte lipid droplet volume

Oocytes were fixed in 4% paraformaldehyde and stored in 1 mg/ml PBS-PVP at 4 °C until staining. Lipid droplets were assessed using BODIPY 493/503 staining (ThermoFisher) followed by confocal microscopy^51^ (see Appendix A.1 for the detailed method).

### Assessment of mitochondrial activity and intracellular ROS

Mitochondrial inner membrane potential (MMP) and ROS level in oocytes were estimated using a combined JC-1 (Invitrogen) and CellROX Deep Red (ThermoFisher) staining, respectively, followed by confocal microscopy^24^ (see Appendix A.2 for the detailed staining method).

### Transmission electron microscopy (TEM)

COCs were fixed in glutaraldehyde solution and processed individually for TEM analysis as described in Appendix A.3. For each COC, images of at least 5 cumulus cells and at least 10 random fields in the oocyte, representative for mitochondria- and ER- rich regions, were acquired at 16500–25000×. Mitochondria in the acquired images were morphologically evaluated by an expert blind to the corresponding treatment group. The dimensions of the mitochondria and ER were also measured using Image-J to determine mitochondrial roundness/elongation and ER swelling among the different treatment groups.

### Quantification of gene expression by qPCR

Total RNA and DNA was extracted from oocytes and cumulus cells using AllPrep DNA/RNA Micro Kit (Qiagen, Venlo, NL) following manufacturers guidelines. Extracted total RNA was treated with RNase–free DNase (Qiagen). The concentration and purity of the isolated RNA samples were determined using a Nanodrop (Thermofisher). Total RNA (50 ng) from each sample was reverse transcribed using SensiScript-RT kit (QIAGEN). Negative-RT control samples (missing reverse transcriptase) were included.

Transcripts of the target genes of interest were quantified by quantitative Polymerase Chain Reaction (Real-Time PCR; qPCR) using SYBR Green (SsoAdvanced Universal SYBR Green supermix, Bio-Rad, Temse, Belgium). Genes of interest were involved in mitochondrial unfolded protein responses (*HSPD1* and *HDSPE1*), mitochondrial biogenesis (*TFAM*), ER stress (*BiP, Atf4* and *Atf6*) and Redox regulation (*SOD2, CAT, PRDX1, PRDX3, PRDX6* and *NRF2*) Quantification was normalized using the geometric mean of 3 housekeeping genes (*18 S, YWHAZ, H2A*) calculated by geNorm software (Camberley, UK). The comparative quantification cycle (Cq) method, ‘2^–ΔΔCq^’, was used to quantify the relative expression level of each gene, as described by Livak and Schmittgen^52^.

### Relative change of mtDNA copy numbers

The ratio of mtDNA to nuclear DNA was determined in each oocyte DNA sample by qPCR of the mitochondrial gene (ND4) and the nuclear gene (bACT). The comparative quantification cycle (Cq) method, ‘2^–ΔΔCq^’, of ND4 vs. bACT was used to quantify the relative mtDNA to nuclear DNA ratio, as described by Livak and Schmittgen^52^.

### Statistical analysis

Statistical analysis was performed with IBM SPSS Statistics 26 (for Windows, Chicago, IL, USA). Numerical data, e.g. blood parameter, MMP, and ROS, were checked for normal distribution and homogeneity of variance. The effect of strain, time and diet and their interaction on weight gain was first checked using Multivariate ANOVA, followed by pairwise comparisons of the diet effect at each time point within each strain. For all the described parameters in serum, oocytes and cumulus cells, a two-tailed independent sample T-test was used to compare HFD and control groups of the same strain, and the control groups of the two strains. Gene expression data were not homogenous and were analyzed using non-parametric independent sample Mann-Whitney test. Categorical data, e.g. proportions of different ultrastructural classifications, were analyzed using Chi square test. The numbers of replicates and oocytes used to generate the data are described in the results section for each parameter. Differences with *P-*values ≤ 0.05 are statistically significant. Differences with *P* values> 0.05 and ≤ 0.1 are not statistically significant and reported as *tendencies*. Data are expressed as means ± S.E.M unless otherwise stated.

## Supplementary information

Supplementary Information.

Supplementary Information 2.

Supplementary Information 3.

Supplementary Information 4.
