# Supplementary material for: Differential effects of high fat diet-induced obesity on oocyte mitochondrial functions in inbred and outbred mice
Source: Sci Rep. 2020 Jun 17;10:9806. doi: 10.1038/s41598-020-66702-6 (PMC7299992; doi:10.1038/s41598-020-66702-6)
Supplement: Supplementary file 1 — Supplementary Information. [file 41598_2020_66702_MOESM1_ESM.docx]

**Title: Differential effects of high fat diet-induced obesity on oocyte mitochondrial functions in inbred and outbred mice.**

Waleed F.A. Marei^a,bŦ*^, Anouk Smits^aŦ^, Omnia Mohey-Elsaeed^c,d^, Isabel Pintelon^d^ , Daisy Ginneberge^e,f^, Peter EJ Bols^a^, Katrien Moerloose^e,f^, Jo L.M.R Leroy^a^

^a^ Gamete Research Centre, University of Antwerp, 2610 Wilrijk, Belgium.

^b^ Department of Theriogenology, Faculty of Veterinary Medicine, Cairo University, Giza 12211, Egypt.

^c^ Department of Cytology and Histology, Faculty of Veterinary Medicine, Cairo University, Giza 12211, Egypt.

^d^ Laboratory of Cell Biology & Histology, University of Antwerp, 2610 Wilrijk, Belgium

^e^ VIB Center for Inflammation Research, Ghent, Belgium

^f^ Department of Biomedical Molecular Biology, Ghent University, Ghent, Belgium

Ŧ W.F.A.M and A.S equally contributed to this study.

*Corresponding author: [Waleed.Marei@uantwerpen.be](mailto:Waleed.Marei@uantwerpen.be)

**Supplementary table 1**. Proportions of different categories of normal and abnormal mitochondrial ultrastructure in oocytes collected from Swiss and B6 mice fed a control or a high fat diet (HFD) for 13 w.

|  | **Swiss Control** | **Swiss HF** | **B6 Control** | **B6 HF** |
| --- | --- | --- | --- | --- |
| **Total** | 229 | 188 | 344 | 746 |
| ***Normal mitochondria*** | ***206 (89.95%)*** | ***82 (43.61%)*** | ***177 (51.45%)*** | ***314 (42.09%)*** |
| a. Spherical | 164 (71.6%) | 63 (33.5%) | 91 (26.5%) | 185 (24.8%) |
| b. Regular Vacuoles | 42 (18.3%) | 19 (10.1%) | 86 (25.0%) | 129 (17.3%) |
| ***Abnormal mitochondria*** | ***23 (10.04%)*** | ***106 (56.38%)*** | ***167 (48.54%)*** | ***432 (57.90%)*** |
| c. Membranous Vacuoles* | 12 (5.24%) | 22 (11.7%) | 63 (18.3%) | 168 (22.5%) |
| d. Electron dense foci | 00 (0%) | 15 (7.98%) | 41 (11.9%) | 106 (14.2%) |
| e. Dumbbell-shaped | 01 (0.44%) | 08 (4.26%) | 16 (4.65%) | 27 (3.62%) |
| f. Elongated | 06 (2.62%) | 26 (13.8%) | 24 (6.97%) | 33 (4.42%) |
| g. Rose petal-shaped | 02 (0.87%) | 14 (7.45%) | 19 (5.52%) | 35 (4.69%) |
| h. Degenerated | 02 (0.87%) | 21 (11.2%) | 04 (1.16%) | 63 (8.45%) |

*Vacuoles containing loose membranous structures. Letters a-h correspond to the representative images shown in supplementary figure 1.
