# Supplementary figures and images for: Differential effects of high fat diet-induced obesity on oocyte mitochondrial functions in inbred and outbred mice
Source: Sci Rep. 2020 Jun 17;10:9806. doi: 10.1038/s41598-020-66702-6 (PMC7299992; doi:10.1038/s41598-020-66702-6)

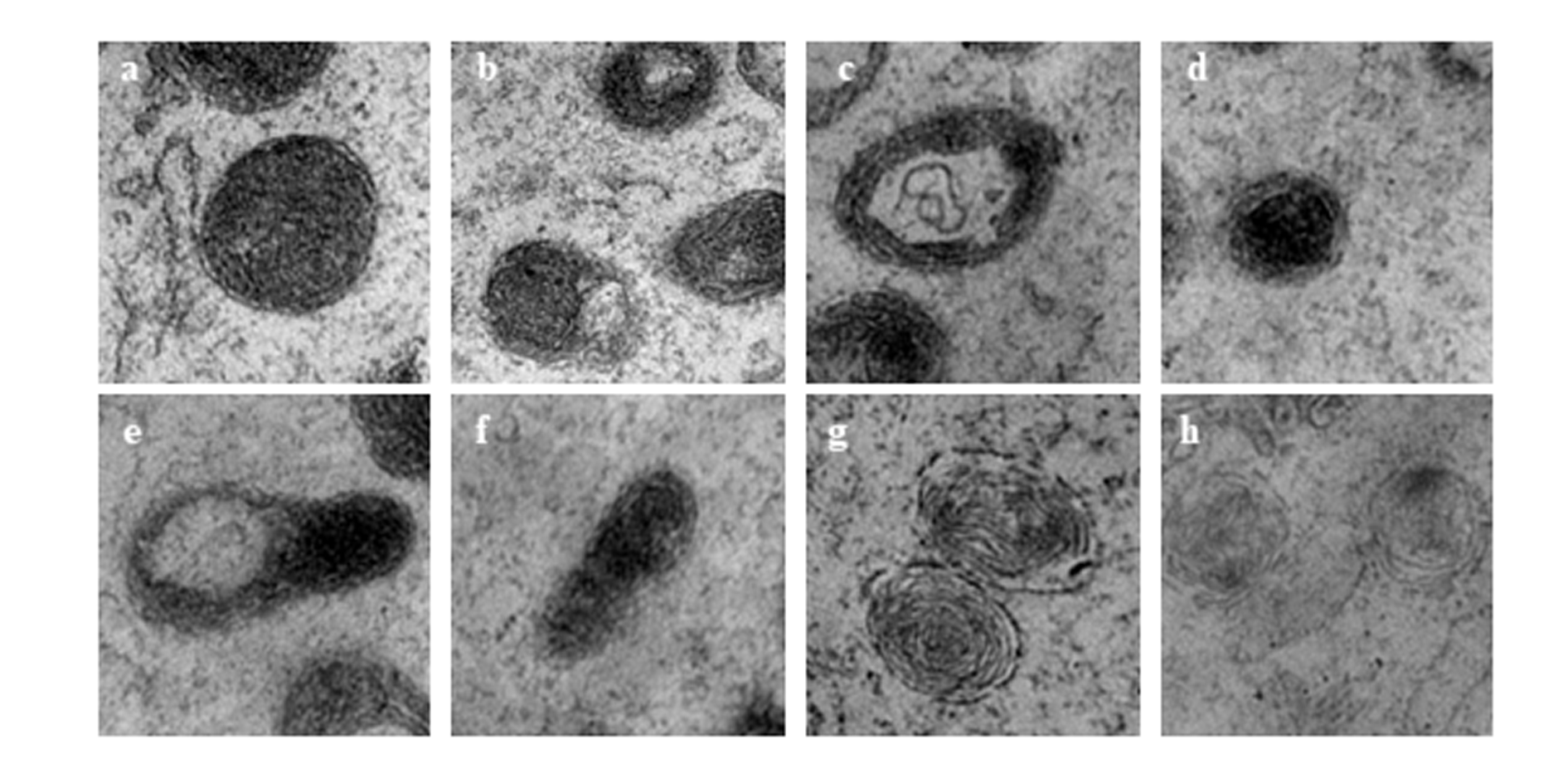

Supplement: Supplementary file 2 — Supplementary Information 2. [file 41598_2020_66702_MOESM2_ESM.tif]
