# Supplementary material for: Differential effects of high fat diet-induced obesity on oocyte mitochondrial functions in inbred and outbred mice
Source: Sci Rep. 2020 Jun 17;10:9806. doi: 10.1038/s41598-020-66702-6 (PMC7299992; doi:10.1038/s41598-020-66702-6)
Supplement: Supplementary file 3 — Supplementary Information 3. [file 41598_2020_66702_MOESM3_ESM.docx]

**Title: Differential effects of high fat diet-induced obesity on oocyte mitochondrial functions in inbred and outbred mice.**

Waleed F.A. Marei^a,bŦ*^, Anouk Smits^aŦ^, Omnia Mohey-Elsaeed^c,d^, Isabel Pintelon^d^ , Daisy Ginneberge^e,f^, Peter EJ Bols^a^, Katrien Moerloose^e,f^, Jo L.M.R Leroy^a^

^a^ Gamete Research Centre, University of Antwerp, 2610 Wilrijk, Belgium.

^b^ Department of Theriogenology, Faculty of Veterinary Medicine, Cairo University, Giza 12211, Egypt.

^c^ Department of Cytology and Histology, Faculty of Veterinary Medicine, Cairo University, Giza 12211, Egypt.

^d^ Laboratory of Cell Biology & Histology, University of Antwerp, 2610 Wilrijk, Belgium

^e^ VIB Center for Inflammation Research, Ghent, Belgium

^f^ Department of Biomedical Molecular Biology, Ghent University, Ghent, Belgium

Ŧ W.F.A.M and A.S equally contributed to this study.

*Corresponding author: [Waleed.Marei@uantwerpen.be](mailto:Waleed.Marei@uantwerpen.be)


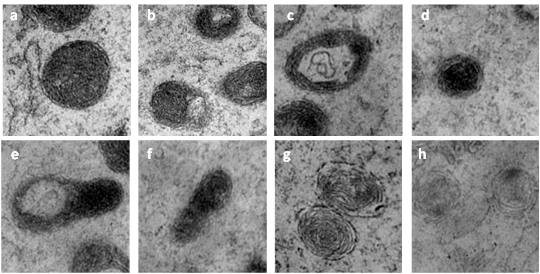


**Supplementary figure 1.** Different forms of mitochondrial ultrastructure that were observed in mouse oocytes. Mitochondrial structure was considered normal when spherical (a) or spherical with regular vacuoles (b). Mitochondrial abnormalities include vacuolation with loose inner membrane structures (c), electron dense foci (d), dumbbell shapes (e), elongation (f), rose petal appearance (g) or degeneration (h).
