# Supplementary material for: Differential effects of high fat diet-induced obesity on oocyte mitochondrial functions in inbred and outbred mice
Source: Sci Rep. 2020 Jun 17;10:9806. doi: 10.1038/s41598-020-66702-6 (PMC7299992; doi:10.1038/s41598-020-66702-6)
Supplement: Supplementary file 4 — Supplementary Information 4. [file 41598_2020_66702_MOESM4_ESM.docx]

**Title: Differential effects of high fat diet-induced obesity on oocyte mitochondrial functions in inbred and outbred mice.**

Waleed F.A. Marei^a,bŦ*^, Anouk Smits^aŦ^, Omnia Mohey-Elsaeed^c,d^, Isabel Pintelon^d^ , Daisy Ginneberge^e,f^, Peter EJ Bols^a^, Katrien Moerloose^e,f^, Jo L.M.R Leroy^a^

^a^ Gamete Research Centre, University of Antwerp, 2610 Wilrijk, Belgium.

^b^ Department of Theriogenology, Faculty of Veterinary Medicine, Cairo University, Giza 12211, Egypt.

^c^ Department of Cytology and Histology, Faculty of Veterinary Medicine, Cairo University, Giza 12211, Egypt.

^d^ Laboratory of Cell Biology & Histology, University of Antwerp, 2610 Wilrijk, Belgium

^e^ VIB Center for Inflammation Research, Ghent, Belgium

^f^ Department of Biomedical Molecular Biology, Ghent University, Ghent, Belgium

Ŧ W.F.A.M and A.S equally contributed to this study.

*Corresponding author: [Waleed.Marei@uantwerpen.be](mailto:Waleed.Marei@uantwerpen.be)

**Appendix: Detailed methods used for assessment of oocyte quality**

**1. Assessment of oocyte lipid droplet volume using Bodipy staining**

Oocytes were permeabilized for 30 min in PBS containing 0.1 % (w/v) saponin (Fiers, Kuurne, Belgium) and 0.1 M glycine. Nuclei were counterstained with 5 μg/ml DAPI (Sigma-Aldrich) for 10 min and oocytes were subsequently washed twice in PBS-PVP. Lipid droplets were stained with 20 μg/ml BODIPY 493/503 (Thermo Fisher Scientific) in PBS for 1h. Oocytes were washed twice in 3 mg/mL PBS-PVP after each step in the staining procedure. Finally, the oocytes were transferred to droplets of 3 mg/ml PBS-PVP on glass-bottom dishes and immediately examined under a confocal microscope. High resolution images were obtained using a Nikon Eclipse Ti-E inverted microscope attached to a microlens-enhanced dual spinning disk confocal system (UltraVIEW VoX; PerkinElmer, Zaventem, Belgium) equipped with 405 and 488 nm diode lasers for excitation of blue and green fluorophores, respectively. For each oocyte, a z-stack of 40 μm (1 μm interval) was taken in the lower half of the oocyte (closest to the objective lens where is the image is sharpest). Images were analyzed using Volocity 6.0.1 software (PerkinElmer) to evaluate the relative differences in lipid droplet content among oocytes in different groups. To avoid noise from background staining, droplets were only included in the analysis if their size was ≥0.5 μm³.

**2. Assessment of mitochondrial activity and intracellular ROS**

Freshly collected oocytes were incubated for 30 min in L15 medium containing JC1 (5,5', 6,6'-tetrachloro-1,1',3,3'-tetraethyl- benzimidazolyl-carbocyanine iodide, 5 µg/mL) and CellRox deep red (2.5 mM) (from 1000X stock solutions in DMSO) at 6 % CO2 and 37°C. They were then washed and transferred to equilibrated L15 medium droplets under mineral oil on a 35 mm dish with a glass bottom. Stained oocytes were immediately examined under a Leica SP8 confocal microscope enclosed in a humid warm chamber (37°C) and equipped with white laser source (Leica WLL) lasers at excitation/emission 488/525 nm (to detect JC-1 monomers or less active mitochondria; green), 561/590 nm (to detect JC1-aggregates or active mitochondria; yellow) and 644/665 nm (to detect CellRox or OS; red). One optical section was examined for each oocyte at the pericortical level. The grey scale intensity in each channel was measured using Leica Application Suite X (LAS X) software. MMP was calculated as the ratio between the grey scale intensity at 590 nm and that at 525nm. ROS level was estimated as the grey scale intensity at 665 nm.

**Transmission electron microscopy (TEM)**

Whole COCs were collected and immediately fixed in 0.1 M sodium cacodylate-buffered (pH 7.4) 2.5 % glutaraldehyde solution at RT. Before processing, individual COCs were embedded in 2% agarose blocks for further handling. Afterwards, blocks were washed three times in 0.1 M sodium cacodylate-buffered (pH 7.4) 7.5 % saccharose solution. Post-fixation was performed by incubating the blocks for 2 h with 1 % OsO4 solution. After dehydration in an ethanol gradient, samples were embedded in EM-bed812. Ultrathin sections were stained with lead citrate, and examined in a Tecnai G2 Spirit Bio TWIN microscope (Fei, Europe BV, Zaventem, Belgium) at 120 kV.
